# Overview of the potential use of fluvoxamine for COVID-19 and long COVID

**DOI:** 10.1007/s44192-023-00036-3

**Published:** 2023-03-21

**Authors:** Kenji Hashimoto

**Affiliations:** Division of Clinical Neuroscience, Chiba University Center for Forensic Mental Health, 1-8-1 Inohana, Chiba, 260-8670 Japan

**Keywords:** Antidepressants, COVID-19, Fluvoxamine, Post-acute COVID-19 syndrome, Serotonin, Sigma-1 receptor

## Abstract

Coronavirus disease 2019 (COVID-19) has presented a serious worldwide threat to public health since its emergence in late 2019. From a safety point of view, drug repurposing has received particular attention. Several clinical studies have demonstrated that the use of fluvoxamine, a selective serotonin reuptake inhibitor with potent sigma-1 receptor agonism, in the early-stage of infection might be associated with the prevention of clinical deterioration in individuals with SARS-CoV-2 infection, although several reports have shown that a low dose of fluvoxamine may be ineffective. There is increasing evidence that SARS-CoV-2 can cross the blood–brain barrier, resulting in a number of psychiatric and neurologic symptoms in COVID-19 survivors. Importantly, about half of COVID-19 survivors experience a variety of long-term sequelae, including psychiatric and neurologic symptoms, known as long COVID. In this priority review, the author presents an overview of the potential use of fluvoxamine in the treatment of COVID-19 and long COVID.

## Introduction

The coronavirus disease 2019 (COVID-19) pandemic has seriously affected public health worldwide since the first report of severe acute respiratory syndrome coronavirus 2 (SARS-CoV-2) infection in Wuhan city, China, in December 2019. Vaccines for SARS-CoV-2 are crucial in preventing the spread of the virus and avoiding clinical deterioration in infected individuals. However, there are some safety concerns regarding COVID-19 vaccines (i.e., risk of myocarditis and pericarditis in young men, risk of immune thrombotic thrombocytopenia, and risk of ischemic stroke in the older population) [1–6]. Furthermore, it seems that immunological imprinting caused by a history of COVID-19 vaccination might modulate the immune response to subsequent infection or vaccination against new SARS-CoV-2 variants [7, 8].

In response to drug safety concerns, drug repurposing has received particular attention [9–15]. Several clinical findings suggest that the use of fluvoxamine, a selective serotonin reuptake inhibitor (SSRI), could be associated with a reduced risk of clinical deterioration in patients with COVID-19. Since the first report by Lenze et al. [16] in November 2020, there have been many studies investigating the effects of fluvoxamine in individuals infected with SARS-CoV-2. However, the efficacy of fluvoxamine for COVID-19 remains controversial.

Infection with SARS-CoV-2 can cause a number of long-term health issues, known as long COVID or post-COVID conditions [17–19], and COVID-19 survivors can experience a variety of sustained sequelae. According to the World Health Organization, long COVID-19 is defined as the symptoms that usually occur after 3 months from the onset of SARS-CoV-2 infection, with symptoms lasting for at least 2 months [20]. Long COVID frequently impacts multiple organs, including respiratory and non-respiratory organs such as the brain [18, 21]. Currently, there are no specific prophylactic or therapeutic drugs to treat long COVID [22].

This review presents an overview of the potential of fluvoxamine in the treatment and clinical management of COVID-19 and long COVID.

## Mechanisms of action of fluvoxamine

### Serotonin transporter

The main target of all SSRIs, including fluvoxamine, is serotonin transporters. Serotonin transporters are localized throughout the body, including in the brain, lungs, and platelets in the blood [11, 23–28]. The order of affinity of SSRIs for human serotonin transporters is paroxetine > sertraline > fluoxetine and escitalopram > citalopram > fluvoxamine (Table 1) [29]. Thus, fluvoxamine is less potent than other SSRIs. Preclinical and clinical data indicate that SSRIs may mediate potent anti-inflammatory activity in mice and patients with depression [30–33]. Although all SSRIs can block serotonin transporters in the body, they do not all produce similar beneficial effects in patients with SARS-CoV-2 infection [34]. Therefore, the inhibition of serotonin transporters may not play a major role in the prophylactic effects of fluvoxamine in patients with COVID-19, although a partial role is possible [11].

**Table 1:** Affinity and pharmacology of SSRIs for serotonin transporter, sigma-1 receptor and action at ASM

SSRIs references	Ki (nM) for serotonin transporter (human)	Ki (nM) for sigma-1 receptor (rat)	Action at sigma-1 receptor (mouse and rat)	Functional inhibitor at ASM
Fluvoxamine	2.3	36^a^ or 17.0^b^	Agonist	Yes
Sertraline	0.26	57^a^ or 31.6^b^	Antagonist	Yes
Fluoxetine	1.1	240^a^ or 191.2^b^	Agonist	Yes
Escitalopram	1.1	288.3^b^	Agonist	Yes
Citalopram	1.6	292^a^ or 403.8^b^	Agonist	Yes
Paroxetine	0.10	1893^a^ or 2041^b^		Yes
References (author/year)	Owen et al. (2001) [29]	^a^Narita et al. (1996) [37] ^b^Ishima et al. (2014) [41]	Hashimoto et al. (2022) [11] Nishimura et al. (2008) [40] Ishima et al. (2014) [41]	Kornhuber et al. (2011) [59] Gulbins et al. (2013) [60] Gulbins et al. (2018) [61]

### Sigma-1 receptor

#### Fluvoxamine as a potent sigma-1 receptor agonist

The sigma-1 receptor, cloned in 1997, contains an endoplasmic reticulum (ER)-retention signal [35]. Hayashi and Su [36] demonstrated that the sigma-1 receptor can function as a novel ER molecular chaperone in cells, and that agonists of the sigma-1 receptor have neuroprotective effects against ER stress associated with systemic inflammation. In 1996, we demonstrated that some SSRIs, including fluvoxamine, sertraline, fluoxetine, and citalopram, have high to moderate affinity to sigma-1 receptors in the rat brain (Table 1) [37]. By contrast, paroxetine had weak affinity to sigma-1 receptor (Table 1). Subsequent studies using mice and a cell culture system showed that three SSRIs (fluvoxamine, fluoxetine, and escitalopram) are agonists of the sigma-1 receptor, whereas sertraline may be an antagonist (Table 1) [38–41]. Among the commercially available SSRIs, fluvoxamine is the most potent agonist for sigma-1 receptor [42–45].

Rosen et al. [46] reported that sigma-1 receptor plays an important role in systemic inflammation using a lipopolysaccharide (LPS)-induced sepsis mouse model. LPS-induced mortality of sigma-1 receptor knockout mice was higher than that of wild-type mice. Blood levels of the proinflammatory cytokines of knockout mice after LPS administration were also higher than those of control mice, suggesting a protective role of sigma-1 receptor in systemic inflammation [46]. Importantly, fluvoxamine could attenuate LPS-induced lethal septic shock in mice [46]. Taken together, these data suggest that sigma-1 receptor may play a crucial role in systemic inflammation, and that activation of sigma-1 receptor by fluvoxamine might protect against ER stress associated with systemic inflammation due to SARS-CoV-2 infection, resulting in reduced mortality [9–11].

#### Sigma-1 receptor in the replication of SARS-CoV-2

Sigma-1 receptor is known to play an important role in the early-stage of viral RNA replication [47], suggesting a key role for sigma-1 receptor in the initial steps of virus-induced host cell reprogramming [11, 48, 49]. Two studies using human protein–protein interaction maps revealed that many compounds that interact with sigma-1 receptor were inhibitors of SARS-CoV-2 replication [50], and that knockdown of *SIGMAR1* caused robust reductions in SARS-CoV-2 replication [51], suggesting a key role of sigma-1 receptor in SARS-CoV-2 replication. These findings suggest that sigma-1 receptor could be a therapeutic target in patients with early-stage COVID-19 [9–11, 52, 53].

### Acid sphingomyelinase (ASM)

Acid sphingomyelinase (ASM) and ceramide are known to play a crucial role in viral infections [11, 54–56]. Since ASM is involved in ceramide generation, the inhibition of ASM could reduce virus entry into epithelial cells. Furthermore, it is suggested that SARS-CoV-2 can activate the ASM/ceramide system, resulting in the formation of ceramide-enriched membrane domains that may facilitate viral entry [11, 57, 58]. All SSRIs, including fluvoxamine, fluoxetine, sertraline, and paroxetine, are shown to inhibit ASM (Table 1), indicating the role of ASM in the pharmacological effects of these SSRIs [59–61].

An observational study in France showed that the use of ASM inhibitors was associated with a reduced risk of intubation or death in severely hospitalized patients with COVID-19 (n = 277) [62]. Among the SSRIs, escitalopram was significantly associated with reduced clinical deterioration in COVID-19 patients, although other SSRIs (i.e., fluoxetine, sertraline, and paroxetine) did not reach statistical significance [62]. Unfortunately, this study did not include fluvoxamine-treated patients. Given the role of ASM and sigma-1 receptor in the entry and replication of SARS-CoV-2 in cells, it is possible that escitalopram may be a potential prophylactic drug for patients with COVID-19 [11].

## Effects of fluvoxamine on COVID-19 patients

In November 2020, Lenze et al. [16] showed that COVID-19 patients (n = 80) treated with fluvoxamine did not undergo clinical deterioration compared with the placebo group (n = 72) (Table 2). In this study (STOP COVID), the patients received a dose of fluvoxamine (50 mg) or a placebo in the evening immediately after baseline assessment and confirmation of eligibility. Then, patients were treated with fluvoxamine (100 mg twice daily for 2 days) or the placebo, as tolerated. Subsequently, fluvoxamine (100 mg 3 times daily) was administered up to day 15. The schedule and dose range of fluvoxamine administration was determined based on the occupancy of sigma-1 receptor in the human brain after oral administration of fluvoxamine [63]. Although this study only involved a small sample size, the results encouraged subsequent non-randomized observational studies and randomized placebo-controlled studies. The same research group performed the STOP COVID 2 study. The patients received a dose of fluvoxamine (50 mg) or a placebo in the evening immediately after baseline assessment. Then, the patients were treated with fluvoxamine (100 mg twice daily) or placebo until day 15 [64]. But this study was terminated early because of futility (Table 2).

**Table 2:** A summary of the randomized clinical trials on the effects of fluvoxamine in patients with COVID-19

Author/year/references	Study name	Country	Number of participants	Dose and duration	Outcome	Efficacy
Lenze et al. (2020) [16]	STOP COVID	USA	Outpatients with COVID-19	Fluvoxamine (50 mg) (n = 80) or placebo (n = 72) was administered in the evening immediately after the baseline assessment. Then, fluvoxamine (100 mg twice daily for two days) or placebo was administered for tolerance, and subsequently increased to a dose of fluvoxamine (100 mg thrice daily) until day 15	Clinical deterioration (1: shortness of breath or hospitalization for shortness of breath or pneumonia and 2: oxygen saturation less than 92% on room air or need for supplemental oxygen to achieve oxygen saturation of 92% or greater) of fluvoxamine group (0/80) was significantly lower than that of placebo group (6/72)	Benefit
Lenze et al. (2021) [64]	STOP COVID 2	USA and Canada	Outpatients with COVID-19	Fluvoxamine (50 mg) (n = 272) or placebo (n = 275) was administered on day 1. Then, fluvoxamine (100 mg twice daily) or placebo was administered as tolerated until day 15	This study was terminated early for futility	No benefit
Reis et al. (2022) [66]	TOGETHER	Brazil	High-risk symptomatic unvaccinated patients with COVID-19	Fluvoxamine (100 mg twice daily) (n = 741) and placebo (n = 756) for 10 days	Fluvoxamine group showed significantly lower proportion of emergency setting for more than 6 h or transferred to tertiary hospital due to COVID-19 compared with placebo group	Benefit
Seo et al. (2022) [70]		Korea	Patients with mild or moderate COVID-19 who were admitted to the community treatment centers	Fluvoxamine (50 mg) (n = 26) or placebo (n = 26) was administered on day 1. Then, fluvoxamine (100 mg twice daily for about 10 days) or placebo was administered for tolerance until discharge from the community treatment centers	A single-blind trial showed that there was no significant differences in clinical deterioration between the fluvoxamine group and placebo group	No benefit
Bramante et al. (2022) [71]	COVID-OUT	USA	Patients with COVID-19	Fluvoxamine (50 mg twice daily) (n = 334) and placebo (n = 327) for 14 days	Fluvoxamine did not prevent the primary events such as hypoxemia, an emergency department visit, hospitalization, or death associated with COVID-19	No benefit
McCarthy et al. (2023) [72]	ACTIV-6	USA	Outpatients with mild to moderate COVID-19	Fluvoxamine (50 mg twice daily) (n = 674) and placebo (n = 614) for 10 days	The median time to sustained recovery was 12 days in the fluvoxamine group and 13 days in the placebo group. There was no significant difference between the two groups	No benefit

Using a prospective observational study, Seftel and Boulware [65] demonstrated the incidence of hospitalization in the fluvoxamine-treated group (0/65) and the observation alone group (6/48) of COVID-19 patients. The incidence of hospitalization was statistically significant difference between the two groups [65]. Reis et al. [66] reported a randomized, placebo-controlled trial of fluvoxamine (100 mg twice daily for 10 days) in unvaccinated adult patients with a risk factor for severe disease progression [66]. The number of patients observed in an emergency room for 6 h or hospitalized in the fluvoxamine group (n = 741) was significantly lower compared with the placebo group (n = 756) (Table 2) [66]. This TOGETHER study suggested that the use of fluvoxamine (100 mg twice daily for 10 days) among early-diagnosed, high-risk, COVID-19 patients could reduce clinical deterioration.

An open-label, prospective cohort trial using intensive care unit patients with COVID-19 in Croatia showed that the mortality rate among the fluvoxamine group (100 mg three times daily for 15 days) (30/51: 58.8%) was significantly lower than that of the matched control group (39/51: 76.5%), although there were no differences in the number of days on ventilator support, the duration of the intensive care unit stays, or the total hospital stay between the two groups [67]. Furthermore, Oskotsky et al. [68] performed a retrospective prospective study of COVID-19 patients treated with SSRIs using electronic health records from healthcare centers (n = 87) in the USA. The relative risk of mortality was significantly lower among COVID-19 patients treated with fluoxetine or fluvoxamine compared with untreated COVID-19 patients. By contrast, there was no significant association between SSRIs (i.e., citalopram, paroxetine, sertraline) other than fluvoxamine or fluoxetine and the risk of death [68]. Moreover, a prospective observational real-world study in Honduras showed that the rates of mortality and hospitalization, and the oxygen requirements of fluvoxamine-treated patients (n = 594), were lower than those of patients not treated with fluvoxamine (n = 63) [69].

Seo et al. [70] reported a single-blind randomized control trial of fluvoxamine (50 mg on day 1 and 100 mg twice daily for 10 days) in adult Korean patients with mild to moderate COVID-19 (Table 2). There was no difference in clinical deterioration between the fluvoxamine group (n = 26) and the placebo group (n = 26). Furthermore, Bramante et al. [71] reported a double-blind, randomized, placebo-controlled trial of fluvoxamine in patients with COVID-19. Fluvoxamine (n = 334, 50 mg twice daily for 14 days) did not prevent the occurrence of primary events such as hypoxemia, an emergency department visit, hospitalization, or death associated with COVID-19 compared with the placebo group (n = 327) (Table 2). In this trial, the authors investigated the effects of other candidates, such as metformin and ivermectin, which did not prevent the occurrence of primary events [71].

In 2023, McCarthy et al. [72] reported the results of the Accelerating COVID-19 Therapeutic Interventions and Vaccines (ACTIV-6) platform randomized clinical trial (Table 2). Outpatients with mild to moderate COVID-19 (n = 1288) were randomly assigned to the fluvoxamine group (50 mg twice daily for 10 days; n = 674) and the placebo group (n = 614). The medial time to sustained recovery was 12 days (interquartile range [IQR], 11–14 days) in the fluvoxamine group and 13 days (IQR, 12–13 days) in the placebo group, with no significant difference between these two groups. The results of this study showed that fluvoxamine did not improve the time to sustained recovery in outpatients with mild to moderate COVID-19 [72].

Recently, two randomized placebo control trials using a large sample size found no evidence to confirm the beneficial effects of fluvoxamine in patients with COVID-19 [71–74]. These two trials used a low dose of fluvoxamine (50 mg twice daily) because of acute common side effects (i.e., nausea). In the treatment of psychiatric disorders (major depressive disorder [MDD], obsessive–compulsive disorder [OCD], social anxiety disorder [SAD], panic disorder), an initial low dose of fluvoxamine (50 mg daily) is used, and then higher doses (100 to 300 mg daily) are used for maintenance. Fluvoxamine has been approved for use in patients with OCD and SAD, but not MDD in the USA. A recent real-world case control study in the USA demonstrated that the odds ratio for acquiring COVID-19 in OCD patients (n = 4558) administered fluvoxamine was lower than that in OCD patients not receiving fluvoxamine (n = 77,511), indicating the protective effects of fluvoxamine in patients with OCD [74]. Considering the maximum dose of fluvoxamine permissible for patients with OCD (300 mg/day), it appears that administration of this drug may prevent clinical deterioration after SARS-CoV-2 infection in OCD patients.

Given the role of sigma-1 receptor in the beneficial effects of fluvoxamine, it seems that a dose of 50 mg twice daily of fluvoxamine is too low for activation of the sigma-1 receptor in the human body. Therefore, the author would like to recommend that high doses of fluvoxamine (100 mg two or three times daily for 10–15 days) may be suitable for patients with early-stage COVID-19. However, further multicenter double-blind, randomized clinical trials with a large sample size of patients administered fluvoxamine (100 mg two or three daily for 10–15 days) are needed.

## Effects of fluvoxamine on long COVID

### Detection of SARS-CoV-2 in the brain

Increasing evidence indicates that SARS-CoV-2 can enter the brain. In 2020, two articles reported the detection of SARS-CoV-2 in the brains of patients who died from COVID-19 [75, 76]. Rhea et al. [77] reported that intravenous or intranasal administration of radio-iodinated S1 protein of SARS-CoV-2 crossed the blood–brain barrier (BBB) in mice by adsorptive transcytosis. It was also reported that SARS-CoV-2 can cross the BBB via a transcellular pathway accompanied by basement membrane disruption without alteration of the tight junctions [78]. Using an in vitro BBB cell culture system, Petrovski et al. [79] demonstrated that the spike protein S1 could cross the human brain endothelial cell barrier. Interestingly, a study using the UK Biobank showed that long-term deleterious effects linked to the olfactory cortex after SARS-CoV-2 infection might be associated with the earliest and most common symptoms, such as loss of smell and taste, among COVID-19 survivors [80, 81]. Furthermore, Song et al. [82] detected SARS-CoV-2 in the cortical neurons of patients who died from COVID-19, and pathological changes in the brain were associated with infection with minimal immune cell infiltrates. In addition, a recent study using autopsies from patients who died from COVID-19 demonstrated that SARS-CoV-2 was widely distributed among multiple respiratory and non-respiratory tissues including the brain [83]. Interestingly, they found few histopathological changes in the brain, despite a substantial viral burden. It is noteworthy that SARS-CoV-2 can produce systemic infection and persist in the body for months [83]. Taken together, these findings indicate that SARS-CoV-2 may enter the brain after infection, resulting in a number of psychiatric and neurologic symptoms [84, 85]. Analysis of the cellular distribution of SARS-CoV-2 and its persistence in the human brain may provide an understanding of the long-lasting psychiatric and neurologic symptoms in COVID-19 survivors. Finally, it is suggested that spike protein, which is derived from SARS-CoV-2 and generated from COVID-19 vaccines, might be able to cross the BBB, resulting in neuroinflammation and blood clots in the brain [86].

**Fig. 1:**
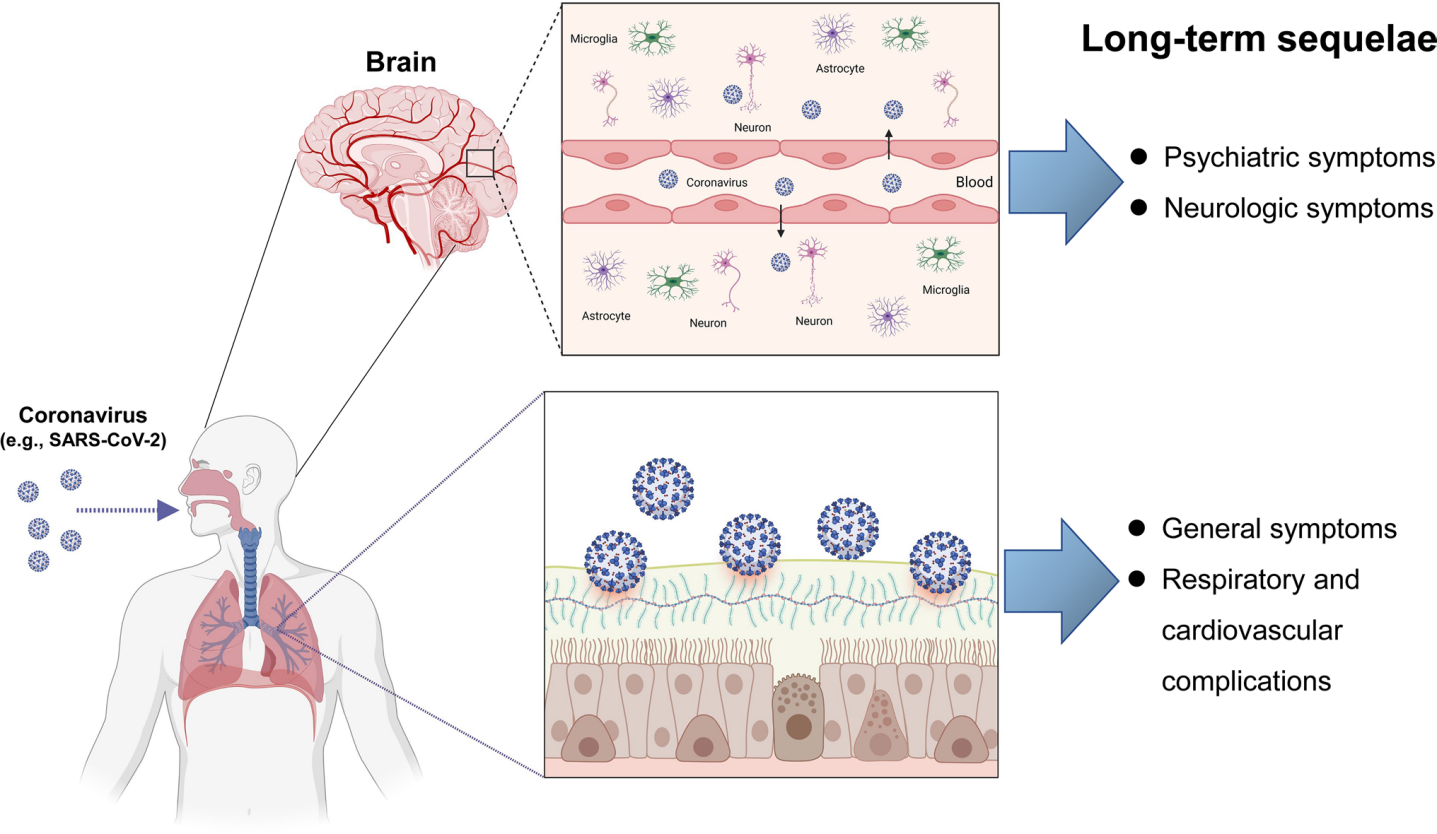
Detection of SARS-CoV-2 in the brain and long-term sequelae in COVID-19 survivors. Coronaviruses, such as SARS-CoV-2, can cause nerve damage through the direct infection pathway (blood circulation and neuronal pathways), and other mechanisms [84]. Several studies using autopsies from patients who died from COVID-19 showed that SARS-CoV-2 was detectable in multiple respiratory and non-respiratory tissues, including the brain [75, 76, 82, 83]. Furthermore, SARS-CoV-2 infection can result in long-term psychiatric symptoms (i.e., depression, anxiety, sleep problems) and neurologic symptoms (i.e., difficulty thinking or concentrating, headaches, dizziness, cognitive impairment) in COVID-19 survivors. The other most common symptoms of long COVID were general symptoms (i.e., tiredness or fatigue), and respiratory and cardiovascular complications (i.e., difficulty breathing or shortness of breath, cough, chest pain, fast-beating or pounding heart). Part of this figure was designed using resources from Biorender.com

Infection with coronaviruses, such as SARS-CoV-2, can cause nerve damage through direct infection pathways (i.e., blood circulation and neuronal pathways), ACE2 (angiotensin-converting enzyme 2), hypoxia, immune injury, and other mechanisms (Fig. 1) [84]. Furthermore, systemic inflammation induced by SARS-CoV-2 infection could contribute to a decrease in neurotrophic factors and monoamines, and activation of microglia in the brain, resulting in long-term psychiatric and neurologic symptoms in COVID-19 survivors [84, 87, 88].

### Effects of SSRIs on long COVID

A systematic review using COVID-19 survivors (n = 250,351) demonstrated that the proportion of COVID-19 survivors experiencing at least one post-acute sequelae of COVID-19 was 54% at 6 months or more (long-term) [89]. The most common symptoms were general symptoms (i.e., tiredness or fatigue), respiratory and heart symptoms (i.e., difficulty breathing or shortness of breath, cough, chest pain, fast-beating or pounding heart), neurologic symptoms (i.e., difficulty thinking or concentrating, headaches, dizziness, cognitive impairment), and psychiatric symptoms (i.e., depression, anxiety, sleep problems) (Fig. 1) [90]. A recent meta-analysis using participants (n = 1,285,407) from 32 countries demonstrated that about half of COVID-19 survivors have a high burden of long-term sequelae in the 12 months after hospital discharge [21]. It is therefore important to treat these long-term sequelae in COVID-19 survivors. However, the precise mechanisms underlying long COVID remain unclear. Unfortunately, there are no potential therapeutic drugs for long-lasting sequelae in COVID-19 survivors, although vaccination has been strongly associated with a low risk of long COVID [91].

A recent retrospective population-based study demonstrated that the use of SSRI at baseline (at or prior to COVID-19 infection) was associated with a significant reduction in the risk of post-acute sequelae of COVID-19 [92]. They found a statistically significant 26% reduction (0.74 [95% CI 0.63–0.88], P = 5 × 10^˗4^) in the relative risk of long COVID among patients (n = 2021) receiving SSRI (i.e., fluvoxamine, fluoxetine, escitalopram, citalopram) with sigma-1 receptor agonism compared with patients (n = 14,584) without SSRI. They also found a statistically significant 25% reduction (0.75 [95% CI 0.62–0.90], P = 0.003) in the relative risk of long COVID among patients (n = 1328) receiving SSRI (i.e., sertraline, paroxetine) without sigma-1 receptor agonism compared with patients (n = 14,584) without SSRI. There were no statistically significant differences in relative risk between the group with SSRI with sigma-1 receptor agonism and the group with SSRI without sigma-1 receptor agonism. In this study, citalopram was included as an SSRI with sigma-1 receptor agonism. However, citalopram did not show agonist activity at the sigma-1 receptor in nerve growth factor-induced neurite outgrowth in PC12 cells [41], suggesting that citalopram should be included as an SSRI without sigma-1 receptor agonism. Therefore, further re-analysis of the data is needed to ascertain the role of sigma-1 receptor agonism on the SSRI-induced significant reduction in relative risk for long COVID. Nonetheless, the current data suggest that baseline use of SSRIs with or without sigma-1 receptor agonism was associated with a significant reduction in the risk of long COVID. In addition, Fenton et al. [93] suggested that SSRIs with anti-inflammatory activity may be effective in the treatment of depression in patients with long COVID.

Khani and Entezari-Maleki [94] reported that fluvoxamine could be a new potential therapeutic drug for long COVID. Considering the crucial role of fluvoxamine for serotonin transport, sigma-1 receptor, and ASM, it is possible that fluvoxamine might possess prophylactic or therapeutic effects in the case of long COVID [95]. Therefore, it may be of interest to investigate whether fluvoxamine could improve persistent symptoms in COVID-19 survivors.

## Limitation

This study has limitation. From the results of several meta-analyses, there is an ongoing debate over the beneficial effects of fluvoxamine for COVID-19 [96–103]. A recent meta-analysis using 6 randomized clinical trials and 5 observational studies demonstrated that the medium dose (100 mg twice daily) of fluvoxamine, but not low dose (50 mg twice daily), was associated with a 21% reduction in the risk of hospitalization, and a 28% reduction in the risk of mortality [104]. A recent prospective interventional open-label cohort study in Uganda demonstrated that the use of fluvoxamine (100 mg twice daily for 10 days) among inpatients with COVID-19 was associated with reduced mortality and increased complete symptom resolution [105]. From the limited placebo-controlled randomized clinical data, we cannot conclude the beneficial effects of fluvoxamine for COVID-19. However, several non-randomized, observational studies suggest that baseline use of SSRIs, with or without sigma-1 receptor agonism, was associated with a reduction in the risk of clinical deterioration after infection and a reduction in the risk of long COVID. Collectively, it seems that fluvoxamine could be a potential candidate to reduce clinical deterioration in COVID-19 patients and long-lasting sequelae in COVID-19 survivors [10, 11, 106, 107].

## Conclusion

As discussed above, it is possible that SARS-CoV-2 can enter the brain and induce neuroinflammation, resulting in acute symptoms (i.e., loss of smell and taste) and long COVID symptoms, including a number of psychiatric and neurologic symptoms. Although the precise mechanisms underlying long COVID remain unclear, invasion of SARS-CoV-2 in the brain may contribute to long-term psychiatric and neurologic symptoms in COVID-19 survivors.

The current clinical data suggest that the use of SSRIs at baseline might be associated with a low risk of subsequent hospitalization by infection and long COVID. Considering the role of sigma-1 receptor in mechanisms of action of SARS-CoV-2 [10, 11], the potent sigma-1 receptor agonist fluvoxamine could be a potential therapeutic or prophylactic drug for COVID-19 and long COVID. Oral use of fluvoxamine has the advantages of a favorable safety profile, it is inexpensive and widely available, and it can be used in children, adolescents, adults, and older individuals [11]. Finally, the author would like to propose that fluvoxamine (i.e., 100 mg two or three times daily for 10–14 days) might be an excellent candidate drug to help deal with a future COVID pandemic.
